# Mode-Locked Er-Doped Fiber Laser by Using MoS_2_/SiO_2_ Saturable Absorber

**DOI:** 10.1186/s11671-019-2888-z

**Published:** 2019-02-19

**Authors:** Lu Li, Ruidong Lv, Zhendong Chen, Jiang Wang, Sicong Liu, Wei Ren, Yonggang Wang

**Affiliations:** 1 School of Science, Xi’an University of Posts and Telecommunications, Xi’an, 710121 China; 2 School of Physics and information Technology, Shaanxi Normal University, Xi’an, 710119 China

**Keywords:** Transition metal dichalcogenides, Nonlinear optical materials, Saturable absorbers

## Abstract

The two-dimensional (2D) layered material MoS_2_ has attracted numerous attentions for electronics and optoelectronics applications. In this work, a novel type of MoS_2_-doped sol-gel glass composite material is prepared. The nonlinear optical properties of prepared MoS_2_/SiO_2_ composite material are measured with modulation depth (ΔT) of 3.5% and saturable intensity (I_sat_) of 20.15 MW/cm^2^. The optical damage threshold is 3.46 J/cm^2^. Using the MoS_2_/SiO_2_ composite material as saturable absorber (SA), a passive mode-locked Er-doped fiber (EDF) laser is realized. Stable conventional soliton mode-locking pulses are successfully generated with a pulse width of 780 fs at the pump power of 90 mW. In the pump power range of 100–600 mW, another stable mode-locking operation is obtained. The pulse width is 1.21 ps and the maximum output power is 5.11 mW. The results indicate that MoS_2_/SiO_2_ composite materials could offer a new way for optical applications.

## Introduction

Nonlinear optical materials, especially those with 2D structures, lay the foundations of optoelectronics development [1–5]. The graphene has been intensively investigated as an optical modulator for use in diverse pulsed lasers and excellent results are obtained [6, 7]. Recently, numerous novel 2D materials such as topological insulators [8, 9], transition metal dichalcogenide (TMD) [10–14], black phosphorus [15], MXene [16], bismuthene [17], metal–organic frameworks [18], and perovskite [19] have demonstrated broadband optical nonlinearities. In addition, these 2D materials are considered as the next generation promising optical modulator materials [20, 21]. The MoS_2_ is a representative TMD semiconductor with crystal layers consisting of three alternating hexagonal planes of Mo and S [22]. Depending on the coordination and oxidation states of transition metal atoms, MoS_2_ can either be semiconducting or metallic in nature. The broadband saturable absorption and high third-order nonlinear susceptibility have been thoroughly studied [23–25]. Recent works demonstrate that the MoS_2_ has better saturable absorption response than graphene by using an open-aperture Z-scan technique for ultrafast nonlinear optical properties [26, 27]. Based on the MoS_2_ materials, the corresponding optical modulator devices have been used for pulsed lasers successfully. So far, pulsed fiber lasers with MoS_2_ at different central wavelengths of 635 nm, 980 nm, 1030 nm, 1560 nm, 1925 nm, and 2950 nm have been achieved [28–33]. Ultrafast fiber lasers based on MoS_2_ emitting pulses with pulse duration from hundreds of femtoseconds to few picoseconds also have been reported [34, 35]. Moreover, high repetition rate pulsed fiber lasers with MoS_2_ have been realized [36, 37].

Usually, MoS_2_ nanomaterials are fabricated via mechanical exfoliation (ME) method [38], liquid phase exfoliation (LPE) method [39], hydrothermal method [40, 41], chemical vapor deposition (CVD) method [42], pulsed laser deposition (PLD) method [43], and magnetron sputtering deposition (MSD) method [44]. Every method has its strengths and weaknesses. For example, ME method is the first reported technique for obtaining layered structure MoS_2_. However, this method has the disadvantages of poor scalability and low yield, hindering the large-scale applications. To overcome the defects of ME method, CVD offers a controllable approach for the production of single and few-layer MoS_2_. While for the MoS_2_ growth, it is often necessary to pretreat of the substrate. PLD and MSD should be the ideal methods for growing high-quality MoS_2_ film directly with different sizes and areas, but with many crystal defects. The reported technology for incorporating MoS_2_ into fiber lasers can be mainly divided into two methods: (1) directly sandwiching the MoS_2_-based SAs between two fiber connectors by mixing the MoS_2_ nanomaterials into polymer film and (2) depositing the MoS_2_ nanomaterials on tapered fiber or D-shaped fiber by using the evanescent wave interaction. The sandwich-type MoS_2_ optical modulators have the advantages of flexibility and convenience. It also has the weak point of low thermal damage. The evanescent wave method can enhance the damage threshold of SAs, but it has the shortcoming of frangibility. For practical applications, tapered fiber or D-shaped fiber-based optical modulators need to be packaged, which makes the fabrication procedure very complicated. Therefore, establishing fine-controlled MoS_2_ nanomaterial still require deeper exploring, and improving effective fabrication method is still a longstanding goal.

In this paper, we demonstrate a novel method to prepare the MoS_2_/SiO_2_ composite materials by doping the MoS_2_ nanomaterials in sol-gel glass. As is well known, the sol-gel method is a mature approach to prepare the glass at low temperature [45, 46]. Doping the MoS_2_ nanomaterials in the sol-gel glass not only has virtues of good antioxidant capacity, but also can effectively increase the mechanical stability. In addition, the sol-gel glass has a good refractive index matching with the optical fiber. Therefore, this type of composite material shows a high environmental damage threshold. By incorporating the proposed MoS_2_/SiO_2_ into EDF laser cavity, we achieve two kinds of mode-locking operation. At the pump power of 90 mW, the conventional soliton mode-locking operation is obtained. The pulse duration is 780 fs. In the pump power range of 100–600 mW, we also realize another stable mode-locking operation. The pulse width is 1.21 ps and the maximum output power is 5.11 mW. The results show that the MoS_2_/SiO_2_ composite materials possess great potential for mode-locked fiber laser applications.

## Methods

### MoS_2_/SiO_2_ Composite Materials Preparation Procedure

The MoS_2_/SiO_2_ composite materials are prepared by the sol-gel method. In the first step, the MoS_2_ dispersion is prepared by liquid-phase exfoliation method. One milligram of MoS_2_ nanosheets is put into the 10 ml deionized water. Then, the MoS_2_ dispersion is ultrasonically for 6 h and the power of ultrasonic cleaner is set as 90 W. After the centrifugation process, we obtain the stable MoS_2_ solution. On the other hand, the tetraethoxysilane (TEOS), ethanol, and deionized water are mixed for the sol-gel glass preparation. In the next step, the MoS_2_ solution and the TEOS mixture are mixed. Then, the MoS_2_ and TEOS mixture is stirred to form the MoS_2_-doped glass. At this time, the hydrochloric acid is added into the obtained mixture to control the PH at low value. Via hydrolysis and polycondensation process, the MoS_2_-doped silica sol is obtained. The hydrolysis and polycondensation process can be described as the following reactions:

$$ \mathrm{nSi}{\left({\mathrm{OC}}_2{\mathrm{H}}_5\right)}_4+{2\mathrm{nH}}_2\mathrm{O}=\mathrm{nSi}{\left(\mathrm{OH}\right)}_4+{4\mathrm{nC}}_2{\mathrm{H}}_5\mathrm{OH}\ \left(\mathrm{hydrolysis}\ \mathrm{reaction}\right) $$

$$ \mathrm{nSi}{\left(\mathrm{OH}\right)}_4={\mathrm{nSiO}}_2+{2\mathrm{nH}}_2\mathrm{O}\ \left(\mathrm{polycondensation}\ \mathrm{reaction}\right) $$

During the hydrolysis process, the alkoxide groups of the TEOS are replaced by the hydroxyl groups. In the polycondensation process, the Si-OH groups produce the Si-O-Si networks. In order to avoid the sol-gel glass cracking and MoS_2_ agglomeration, the MoS_2_-doped silica sol are stirred at 50 °C for 5 h. Then, the MoS_2_-doped silica sol are put into the plastic cells and aged at room temperature for 48 h. In the final step, put the silica sol into a dry box at 60 °C for 1 week to form solid MoS_2_-doped glass.

### Fiber Laser Cavity

**Fig. 1:**
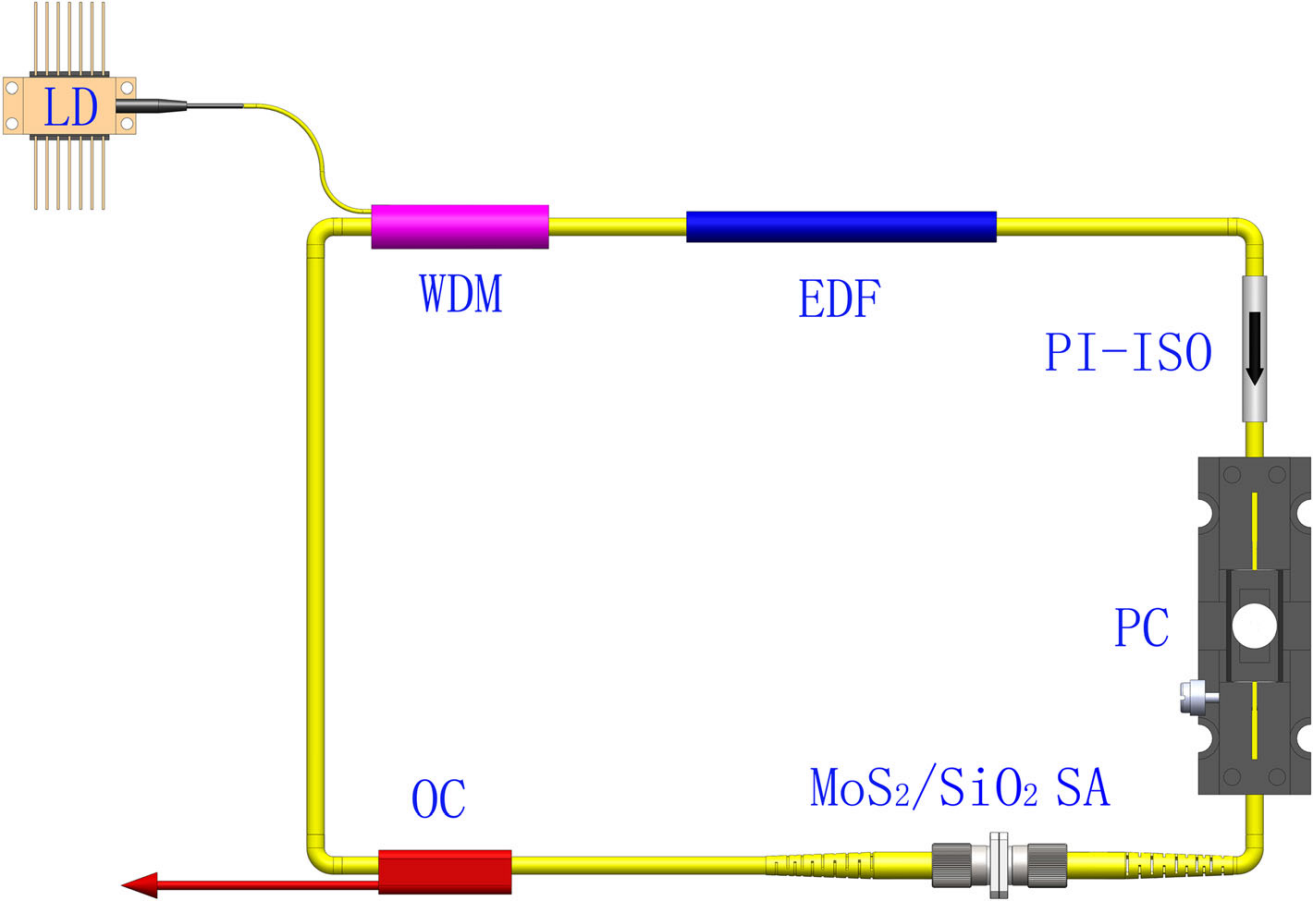
Experimental setup of EDF mode-locked fiber laser

The layout of the EDF laser with MoS_2_/SiO_2_ composite material is displayed in Fig. 1. The ring laser cavity is used. The pump source is a fiber-coupled laser diode (LD) with the maximum output power of 650 mW, which delivers the pump laser into the laser cavity via the wavelength division multiplexer (WDM). A 1.2-m-long EDF is employed as the gain medium. A polarization independent isolator (PI-ISO) is used to ensure the unidirectional operation in the ring laser cavity. A polarization controller (PC) is engaged to achieve different polarization states. A MoS_2_/SiO_2_ composite material is sandwiched between two fiber ferrules. The 10/90 optical coupler is used at the laser cavity output port. The total length of the laser oscillator cavity is about 13.3 m.

## Results and Discussion

### Characterization of MoS_2_/SiO_2_ Composite Materials

**Fig. 2:**
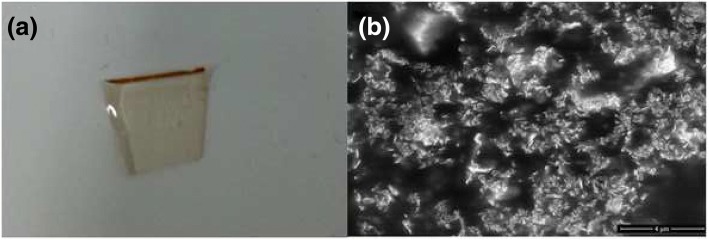
**a** Digital photos. **b** SEM image

**Fig. 3:**
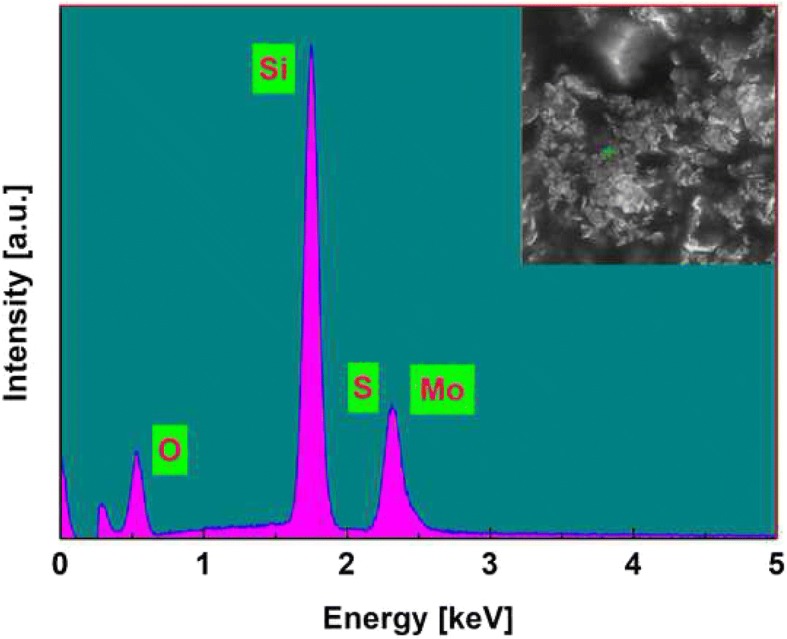
EDS spectrum

**Fig. 4:**
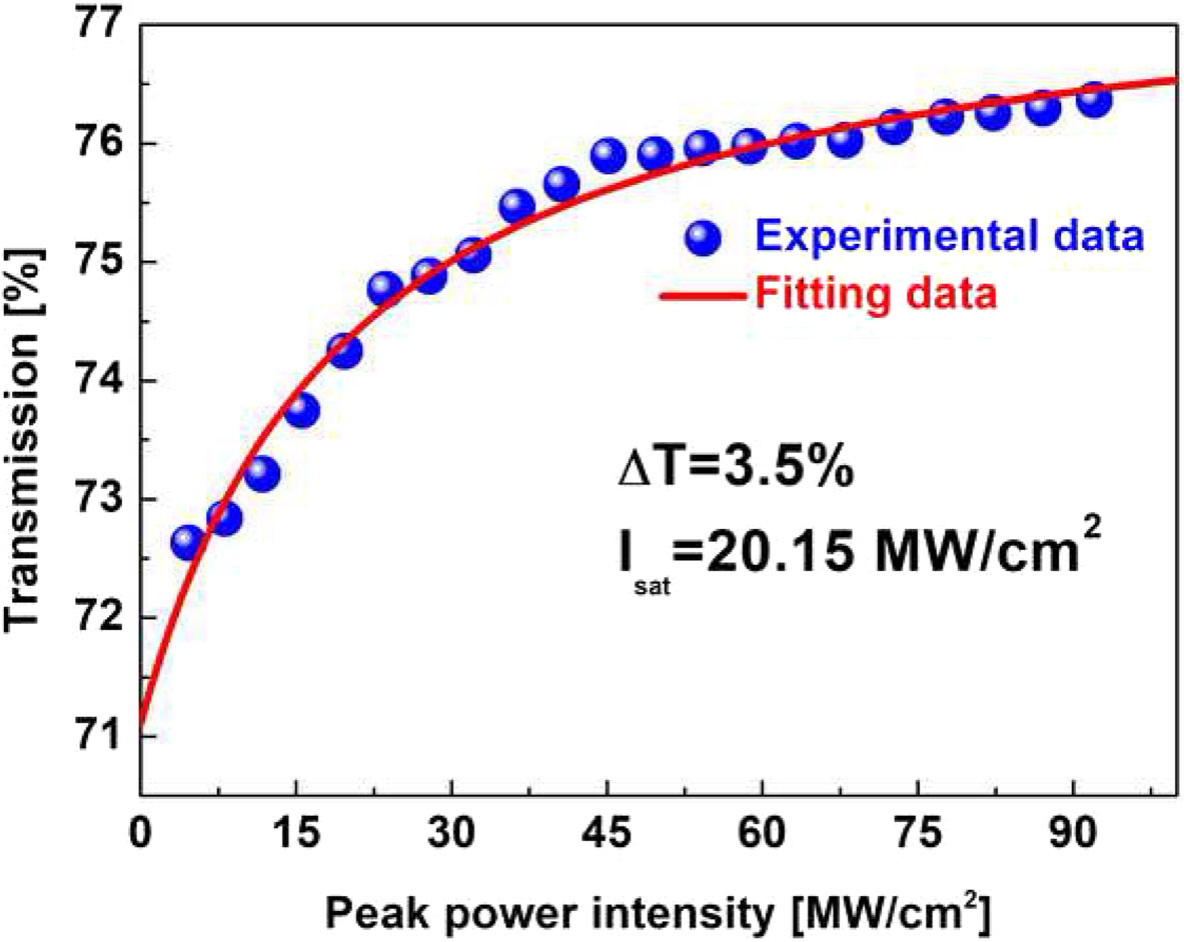
Nonlinear optical properties of MoS_2_/SiO_2_ composite materials

As is shown in Fig. 2a, the prepared MoS_2_/SiO_2_ composite material is the brown color, indicating the MoS_2_ nanosheets are incorporated into the silica glass. Figure 2b shows the SEM image. The MoS_2_/SiO_2_ composite material is also characterized by energy dispersive X-ray spectrometer (EDS). Figure 3 shows the EDS spectrum, which indicates that the prepared MoS_2_/SiO_2_ glass contains three elements (Mo, S, and Si). The nonlinear optical properties of MoS_2_/SiO_2_ glass are investigated by the balanced twin-detector measurement system. The pulse laser source is the home-made EDF fiber laser with a central wavelength of 1550 nm, pulse width of 500 fs, and repetition rate of 23 MHz. As can be seen from Fig. 4, the modulation depth (ΔT) and saturable intensity (I_sat_) are measured to be 3.5% and 20.15 MW/cm^2^, respectively. A femtosecond Ti:sapphire laser (central wavelength 800 nm, pulse width 250 fs, repetition rate 100 kHz) is used as the source to investigate the thermal damage of MoS_2_/SiO_2_ composite material. The optical damage of the MoS_2_/SiO_2_ appears when the test power is adjusted to 3.46 J/cm^2^, which is much higher than that of semiconductor saturable absorber mirror (SESAM) (500 μJ/cm^2^).

### MoS_2_/SiO_2_ Mode-Locking Fiber Laser

**Fig. 5:**
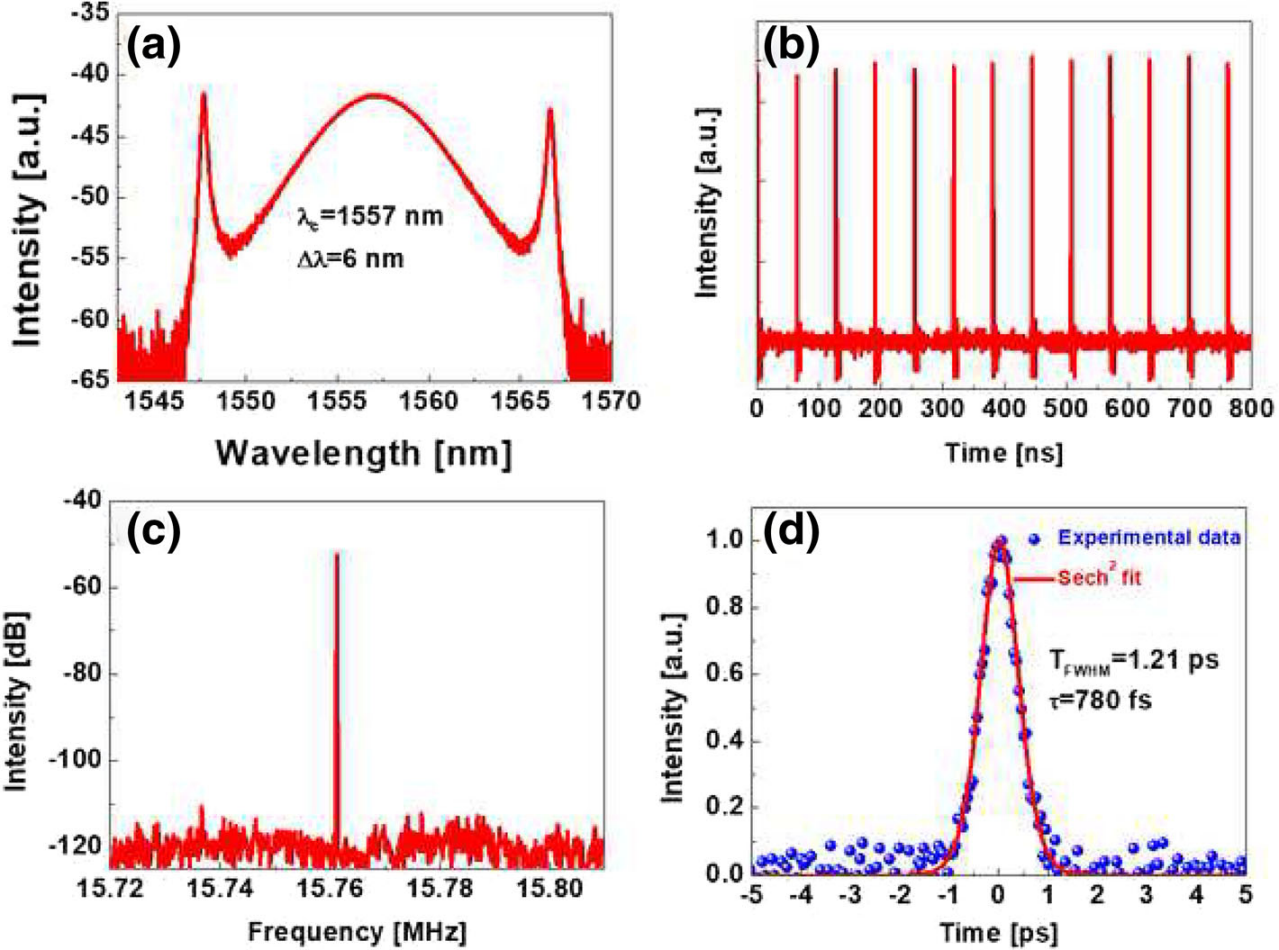
Conventional soliton experimental results: **a** optical spectrum, **b** pulse train, **c** radio-frequency spectrum, **d** autocorrelation trace

The conventional soliton mode-locking experimental results are shown in Fig. 5. The mode-locking operation is observed at the pump power of 90 mW accompanying hysteresis phenomenon [47]. By adjusting the pump power lower to 75 mW, the mode-locking state is still maintained. The optical spectrum of mode-locking pulses at the pump power of 90 mW is depicted in Fig. 5a. The central wavelength is located at 1557 nm and the 3-dB spectral width is 6 nm. It can be seen clearly that the Kelly sidebands appeared at both sides of spectrum symmetrically, indicating the fiber laser works in conventional soliton mode-locking state. Figure 5b shows the performance of the pulse train, which has uniform intensity. The interval of two pulses is 64.2 ns, corresponding to the cavity roundtrip time. To further study the stability of soliton pulse, the radio-frequency spectrum is measured. Figure 5c shows that the fundamental repetition rate is 15.76 MHz and the signal-to-noise ratio (SNR) is 65 dB. The pulse duration is measured by an autocorrelator. Figure 5d shows the autocorrelation curve. The full width at half maximum (FWHM) is measured to be 1.21 ps, indicating the pulse duration is 780 fs if a Sech^2^ fit is used. We just increase the pump power to 100 mW and keep the PC unchanged, the laser enters into multiple pulses operation mode-locking regime, presenting instability and fluctuations, which means the mode-locking operates in narrow pump range.

**Fig. 6:**
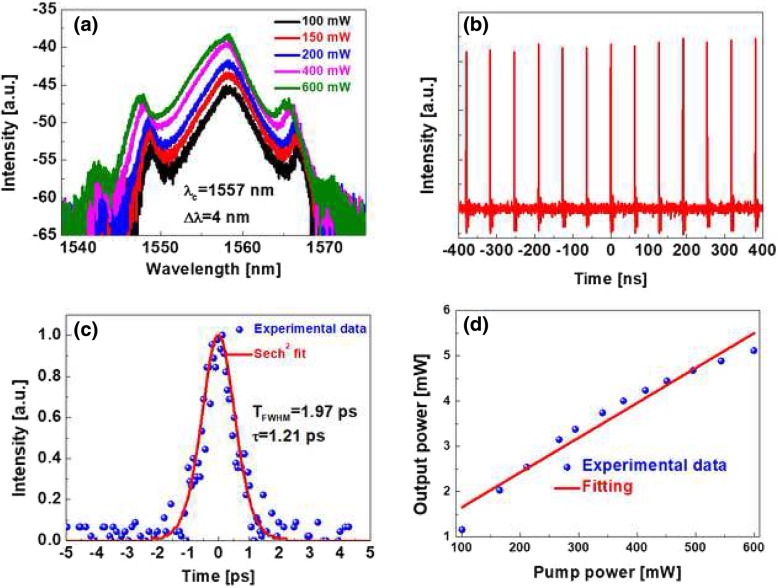
Experimental results: **a** optical spectrum, **b** pulse train, **c** autocorrelation trace, **d** output power

During the experiments, we achieve another mode-locking state. By adjusting the pump power to 100 mW and the PC rotation, we obtain this mode-locking operation state. Figure 6a records the corresponding optical spectrum. The optical spectrum is getting wider and wider with pump power increasing. Gradually increasing the pump power to 600 mW, this mode-locking operation can always be maintained. It is observed that the sides appeared in the optical spectrum with relative small intensity. The central wavelength is 1557 nm and 3-dB spectral width is 4 nm at the pump power of 600 mW. The oscilloscope trace for the mode-locking state is depicted in Fig. 6b; the interval of two pulses is 64.2 ns, verifying that the fiber laser is working in the fundamental mode-locking state. The autocorrelation trace is displayed in Fig. 6(c), the full width at half maximum (FWHM) is 1.97 ps, which means the pulse duration is 1.21 ps if a Sech^2^ fit is used. The average output power characteristics are shown in Fig. 6d. As the pump power increases, the average output power increases almost linearly. The maximum output power is measured to be 5.11 mW at the pump power of 600 mW.

## Conclusion

In conclusion, we have reported the MoS_2_/SiO_2_ composite materials, which are prepared by incorporating the MoS_2_ nanomaterials in sol-gel glass. EDS spectrum identifies the main component of prepared MoS_2_/SiO_2_ glass. The modulation depth and saturable intensity of MoS_2_/SiO_2_ composite materials are measured to be 3.5% and 20.15 MW/cm^2^, respectively. Mode-locked fiber laser with MoS_2_/SiO_2_ is further demonstrated. The conventional soliton mode-locking state with a pulse duration of 780 fs is realized at the pump power of 90 mW. In the pump power range of 100–600 mW, another stable mode-locking state is presented. The pulse width is 1.21 ps and the maximum output power is 5.11 mW. Our results show that the MoS_2_/SiO_2_ composite materials possess a good prospect in ultrafast photonics and the sol-gel method provides a new way for fabrication of TMD optical devices.

## Abbreviations

2D: Two-dimensional
CVD: Chemical vapor deposition
EDF: Er-doped fiber
EDS: Energy dispersive X-ray spectrometer
FWHM: Full width at half maximum
I_sat_: Saturable intensity
LD: Laser diode
LPE: Liquid phase exfoliation
ME: Mechanical exfoliation
MSD: Magnetron sputtering deposition
PC: Polarization controller
PI-ISO: Polarization independent isolator
PLD: Pulsed laser deposition
SA: Saturable absorber
SESAM: Semiconductor saturable absorber mirror
SNR: Signal-to-noise ratio
TEOS: Tetraethoxysilane
TMD: Transition metal dichalcogenide
WDM: Wavelength division multiplexer
ΔT: Modulation depth
